# Enriched Environment Cues Suggest a New Strategy to Counteract Glioma: Engineered rAAV2-IL-15 Microglia Modulate the Tumor Microenvironment

**DOI:** 10.3389/fimmu.2021.730128

**Published:** 2021-09-06

**Authors:** Alessandro Mormino, Giovanni Bernardini, Germana Cocozza, Nicoletta Corbi, Claudio Passananti, Angela Santoni, Cristina Limatola, Stefano Garofalo

**Affiliations:** ^1^Department of Physiology and Pharmacology, Sapienza University, Rome, Italy; ^2^Department of Molecular Medicine, Laboratory Affiliated to Istituto Pasteur Italia, Sapienza University, Rome, Italy; ^3^Instituto di Ricovero e Cura a Carattere Scientifico (IRCCS) Neuromed, Pozzilli, Italy; ^4^Department of Molecular Medicine, CNR-Institute of Molecular Biology and Pathology, Sapienza University, Rome, Italy; ^5^Department of Physiology and Pharmacology, Laboratory Affiliated to Istituto Pasteur Italia, Sapienza University, Rome, Italy

**Keywords:** glioma, enriched environment, microglia/macrophages, NK cell, IL-15, adeno-associated virus

## Abstract

Several types of cancer grow differently depending on the environmental stimuli they receive. In glioma, exposure to an enriched environment (EE) increases the overall survival rate of tumor-bearing mice, acting on the cells that participate to define the tumor microenvironment. In particular, environmental cues increase the microglial production of interleukin (IL)-15 which promotes a pro-inflammatory (antitumor) phenotype of microglia and the cytotoxic activity of natural killer (NK) cells, counteracting glioma growth, thus representing a virtuous mechanism of interaction between NK cells and microglia. To mimic the effect of EE on glioma, we investigated the potential of creating engineered microglia as the source of IL-15 in glioma. We demonstrated that microglia modified with recombinant adeno-associated virus serotype 2 (rAAV2) carrying IL-15 (rAAV2-IL-15), to force the production of IL-15, are able to increase the NK cells viability in coculture. Furthermore, the intranasal delivery of rAAV2-IL-15 microglia triggered the interplay with NK cells *in vivo*, enhancing NK cell recruitment and pro-inflammatory microglial phenotype in tumor mass of glioma-bearing mice, and ultimately counteracted tumor growth. This approach has a high potential for clinical translatability, highlighting the therapeutic efficacy of forced IL-15 production in microglia: the delivery of engineered rAAV2-IL-15 microglia to boost the immune response paves the way to design a new perspective therapy for glioma patients.

## Introduction

Among brain malignancies, glioma is the most diffuse tumor, and its prognosis is significantly correlated with pathological grades. Malignant glioblastoma (GBM) (classified as >IV grade of glioma by the World Health Organization (WHO)) creates an immunosuppressive microenvironment that supports its own growth, leaving few disease-free months to patients (1). The pattern of cytokines produced by glioma and infiltrating cells dramatically alters the tumor-suppressive functions of innate and adaptive immune cells, hampering their antitumor activity (2). Microglia/macrophages (M/M^Φ^) invade glioma with high efficiency, representing up to 30% of tumor mass (3), but are “tricked” to favor tumor growth: tumor-derived cytokines cooperate to polarize M/M^Φ^ toward a pro-tumoral phenotype, characterized by the production of anti-inflammatory, pro-invasive cytokines promoting tumor proliferation, angiogenesis, and invasion. By contrast, M/M^Φ^ with a pro-inflammatory phenotype are endowed with antitumor activities (4).

Consistently to a pro-tumoral microenvironment, also natural killer (NK) cells are silenced by glioma. The maturation and survival of NK cells require IL-15, which also stimulates interferon (IFN)-γ production (5), and mice lacking IL-15 or one of its signaling components are devoid of NK cells (6). Activated NK cells are able to exert antitumor function by direct cytotoxic activity, producing several cytokines and chemokines (7–9). Among the cytokines, IFN-γ contributes to the antitumor activity in different neoplasms by inhibiting tumor cell proliferation and angiogenesis (10, 11). Furthermore, IFN-γ counters the acquisition of a pro-tumor phenotype by M/M^Φ^ and reverses toward a pro-inflammatory phenotype (11, 12). Nevertheless, invasion of glioma by NK cells is weak and conflicting results are reported on the impact of NK cells on GBM clinical outcome (13–15). In addition, the presence of glioma cells attenuates NK cell cytotoxic activity (16).

Over the past few years, many pieces of evidence highlighted the beneficial effects of housing animals in an enriched environment (EE) in physio-pathological conditions, obtained with prolonged voluntary physical exercise, social interactions, and sensory stimulation. EE has been shown to shape neuronal circuits and to reduce the impact of several neurodegenerative diseases and cancers both in animal models and in patients (11, 17–20). In addition, environmental stimuli also shape immune functions, modulating NK cells (20, 21), T helper cells (22), and myeloid cells (23).

We have recently shown that EE exposure reduces the tumor size and proliferation rate of glioma cells, with increased survival in human and murine glioma mouse models (20). Exploring the mechanisms involved in the effects induced by EE, we described an increased production of IL-15 by microglial cells, with effects on NK cell cytotoxicity and IFN-γ production. EE exposure also affects the phenotype of microglia, switching them toward an antitumor state (11). Consistently, we demonstrated that local administration of IL-15 in tumor mass directly modulates glioma microenvironment, re-boosting the immune response against tumor cells and activating the interaction between NK cells and microglia (11).

In this study, we explored the potential use of microglia engineered to express IL-15 upon infection with a recombinant adeno-associated virus serotype 2 (rAAV2) carrying IL-15 (rAAV2-IL-15), to simulate the effect of EE on glioma. AAV are considered safe for human gene therapy and have been successfully used to target several cell types within the central (CNS) and peripheral nervous systems (PNS), including neurons, oligodendrocytes, astrocytes, Müller glia, Schwann cells, and also microglia (24). We demonstrated that rAAV2-IL-15 microglia increase the viability of cocultured NK cells, without affecting their activation state *in vitro*. Furthermore, the intranasal (i.n.) administration of rAAV2-IL-15 microglia to glioma-bearing mice reaches the tumor mass, with effects on the tumor volume, NK cell recruitment, and M/M^Φ^ phenotype.

Altogether, our data support the key role of microglia-derived IL-15 in regulating the glioma microenvironment and boosting immune cell activation.

## Materials and Methods

### Materials

All culture media, fetal bovine serum (FBS), goat serum, penicillin G, streptomycin, glutamine, Na pyruvate, ThermoScript RT-PCR Systems, Hoechst (#33342, RRID : AB_10626776), and mouse anti-IL-15 (PA5-47014) were from Gibco Invitrogen (Carlsbad, CA, USA). Glucose, hematoxylin, eosin, Percoll, Papain (#P3125), phosphate buffered-saline (PBS) tablet (#P4417), bovine serum albumin (BSA), and deoxyribonuclease I were from Sigma-Aldrich (Milan, Italy). NKp46 (M20) (#sc-18161, RRID: AB_2149152) antibody (Ab) and Arg1 (E-2) (#sc-271430) were from Santa Cruz Biotechnology (Santa Cruz, CA, USA). Secondary Abs were from DAKO (Milan, Italy). Rabbit anti-Iba1 was from Wako (VA, USA). Anti-mouse (clone name in brackets) CD45 (104), CD69 (H1.2F3), CD3 (145-2C11), NKG2D (CX5), and NK1.1 (Pk136) mAbs directly conjugated to FITC, PE, PerCP 5.5, allophycocyanin, and allophycocyanin-eFluor 780 were from Ebioscences (Thermo Fisher Scientific, Waltham, MA USA). The pAAV-CMV-IL-15-GFP vector (CW304678) expressing the mouse IL-15 ORF under the control of a Cytomegalovirus promoter was from Tema Ricerca (Bologna, Italy).

### Mice and Cell Lines

The experiments described in the present work were approved by the Italian Ministry of Health in accordance with the guidelines on the ethical use of animals from the European Community Council Directive of September 22, 2010 (2010/63/EU). We used C57BL/6 mice from Charles River Laboratories (Calco, Italy). The heterozygous Cx3cr1^+/GFP^ was from The Jackson Laboratory. We always used 2-month-old male mice.

The GL261 glioma cell line (RRID : CVCL Y003) was cultured in DMEM supplemented with 20% heat-inactivated FBS, 100 IU/ml penicillin G, 100 µg/ml streptomycin, 2.5 µg/ml amphotericin B, 2 mM glutamine, and 1 mM sodium pyruvate. Primary murine microglia were cultured in DMEM supplemented with 10% FBS.

### Production, Purification, and Titration of Recombinant AAV2 Stocks

AAV-293 cell lines were grown in DMEM (Gibco-BRL, Grand Island, NY, USA) supplemented with 10% inactivated fetal bovine serum (Gibco-BRL), L-glutamine, and penicillin/streptomycin. For rAAV2-IL-15 and rAAV2-GFP viral stock productions, AAV-293 cells were transfected with calcium phosphate-based protocol using the “AAV Helper-Free System” (StrataGene) (25). For each AAV stock, about 30 10-cm dishes were transfected with pAAV-CMV-IL-15-GFP plasmid (or pAAV-CMV-GFP), pAAV-RC plasmid, and pAAV-helper plasmid (10 µg of each plasmid for each dish). All the AAV transduction experiments were carried out using serotype 2. AAV2 viral particles were purified from DMEM growth medium supernatant 72 h after transfection. The cell growth medium with virus suspension was extensively centrifuged, concentrated 20:1, and dialyzed twice against a physiological solution for 5–6 h. The virus titer was quantified by real-time PCR (v.p/ml) as previously described (26). The virus titer obtained was 2–5 × 10^11^ v.p./ml for each stock preparation. AAV-293 (as control, data not shown) or microglia were incubated for 7 days before analysis. Serial dilutions of rAAV2-GFP or rAAV2-IL-15 in the growth medium were tested to achieve maximal infectivity (in microglia and microglia/NK) with no significant cell death.

#### Primary Microglial Cultures and Microglial Infection

Microglial cultures were obtained from mixed glial cultures derived from the cerebral cortices of postnatal day 0–1 (P0–P1) C57BL/6 mice, as described in [20]. In brief, cortices were chopped and digested in 15 U/ml papain for 20 min at 37°C. Cells (5 × 10^5^ cells/cm^2^) were plated on flasks coated with poly-L-lysine (100 mg/ml) in DMEM supplemented with 10% FBS, 100 U/ml penicillin, and 0.1 mg/ml streptomycin. After 7–9 days, cells were shaken for 2 h at 37°C to detach and collect microglial cells. These procedures gave almost pure microglial cell populations. The remaining attached cells were astrocytes. Microglia (18×10^4^) were incubated for 7 days in 24 wells with serial dilutions of rAAV2-GFP or rAAV2-IL-15.

#### Coculture of Primary Microglia–NK Cells

To analyze NK cell degranulation, purified NK cells from the spleen of C57BL/6 mice (using Miltenyi MACS Isolation Kit #130-115-818) were activated O/N in IL-15 (50 ng/ml) and then coincubated with primary culture of microglia infected with rAAV2-GFP/IL-15 for 7 days for 4 h at the ratio 1:1. NK cells were collected from the non-adherent fraction and analyzed by FACS.

### Flow Cytometry FACS

NK cells were collected from the microglial coculture and stained for 20 min with fluorochrome-conjugated antibodies. All cells were analyzed by flow cytometry using FACSCanto II (BD Biosciences), and data were elaborated using FlowJo Version 9.3.2 software (TreeStar).

### Intracranial Injection of Glioma

Male C57BL/6 mice were anesthetized with chloral hydrate (400 mg/kg, i.p.) and placed in a stereotaxic head frame. Animals were stereotactically injected with 7.5 × 10^4^ GL-261: a median incision of ~1 cm was made, a burr hole was drilled in the skull, and cells were injected 2 mm lateral (right) and 1 mm anterior to the bregma in the right striatum. Cell suspensions in PBS (4 µl) were injected with a Hamilton syringe at a rate of 1 ml/min at a 3-mm depth. After 17 days, animals were sacrificed for different analyses.

### Intranasal Microglial Delivery

Starting at 7 days after glioma injection, mice were randomly grouped for the treatments. Thirty minutes before the treatment, mice were anesthetized using isofluorane and treated with 6 µl of hyaluronidase from bovine testes 10 mg/ml per nostril. Then, mice were anesthetized as described before and rAAV2-GFP- and rAAV2-IL-15-infected microglia and saline solution were intranasally administrated at the concentration of 1×10^6^ cells/24 µl of saline solution. Mice were treated with 24 µl of the three treatments at a rate of 6 µl per nostril every 2 min after 7 and 10 days from gliomal inoculation.

#### Histopathological Evaluation of Tumor Volume

After 17 days from glioma cell injection, brains were isolated for morphological evaluation of tissues and fixed in 4% buffered formaldehyde. Coronal brain sections (20 µm) were prepared by standard procedures and stained with hematoxylin and eosin. A section every 100 µm was collected, and the tumor area was evaluated using ImageTool 3.00.

#### Immunostaining

Seventeen days after injection of GL261 cells, mice were sacrificed and the brains fixed in 4% formaldehyde. Subsequently, the brains were incubated in 30% sucrose solution for 48 h and then snap frozen. Cryostat sections (10 µm) were boiled for 10 min in citrate buffer, pH 6.0, at 95°C–100°C, then rinsed in PBS and incubated with 3% goat serum in 0.3% Triton X-100 for 1 h at room temperature and then overnight at 4°C with specific antibodies in PBS containing 1% goat serum and 0.1% Triton X-100. The sections were stained with the following primary Abs: anti-IL-15 (1:100), anti-Iba1 (1:500), anti-NKp46 (1:50), and anti-Arg1 (1:500). After several washes, sections were stained with the fluorophore-conjugated antibody and Hoechst (1:3,000) for nucleus visualization and analyzed by fluorescence microscopy.

#### Image Acquisition and Data Analysis

Images were digitized using a CoolSNAP camera (Photometrics) coupled to an ECLIPSE Ti-S microscope (Nikon) and processed using MetaMorph 7.6.5.0 image analysis software (Molecular Devices). Brain slices were scanned by consecutive fields of vision (×10 objective lens) to build a single image per section. The percentage of positive cells was measured as the ratio of the area occupied by fluorescent cells versus the total tumor area (by converting pixels to square millimeters). For comparison between different treatments, at least 12 coronal sections per brain around the point of injection were analyzed.

### Morphological Analysis of Iba1+ Cells

Brain slices from perfused GL261-beraing mice mice were analyzed by confocal microscopy and skeleton analysis to assess Iba1+ cell morphology. Twenty-millimeter z-stacks were acquired at 0.5-µm intervals using an FV1000 laser scanning microscope (Olympus) at ×60 objective. Maximal intensity projections of each image were generated, binarized, and skeletonized using the Skeletonize 2D/3D plugin in ImageJ, after which the Analyze Skeleton plugin (http://imagej.net/AnalyzeSkeleton) was applied. The average branch number (process end points per cell) and length per cell were recorded for each image with a voxel size exclusion limit of 150 applied. The number of single- and multiple-junction points was additionally calculated to give an indication of branching complexity. The areas of the soma and scanning domain were measured for each cell. Acquisition files were then processed with ImageJ software for two-dimensional analysis.

#### Environmental Enrichment

Mice were housed for 5 weeks in EE: 10 mice for cage (36 cm × 54 cm × 19 cm), in the presence of an assortment of objects, including climbing ladders, seesaws, running wheel, balls, plastic and wood objects suspended from the ceiling, paper, cardboard boxes, and nesting material. Toys were changed every 2 days, and the bedding every week. Control SE mice were in pair, in standard cages: 30 cm × 16 cm × 11 cm.

#### Real-Time PCR

Primary microglial cells, obtained from pups (P0–2) incubated for 7 days with vehicle, rAAV2-GFP, and rAAV2-IL-15 at different dilutions, were lysed in TRIzol reagent for isolation of RNA. Reverse transcription reaction was performed in a thermocycler (MJ Mini Personal Thermal Cycler; Bio-Rad) using iScript™ Reverse Transcription Supermix (Bio-Rad) according to the manufacturer’s protocol, under the following conditions: incubation at 25°C for 5 min, reverse transcription at 42°C for 30 min, and inactivation at 85°C for 5 min. Real-time PCR (RT-PCR) was carried out in an iCycler IQ Multicolor RT-PCR Detection System (Bio-Rad) using SsoFast EvaGreen Supermix (Bio-Rad) according to the manufacturer’s instructions. The PCR protocol consisted of 40 cycles of denaturation at 95°C for 30 s and annealing/extension at 60°C for 30 s. For quantification analysis, the comparative threshold cycle (Ct) method was used. The Ct values from each gene were normalized to the Ct value of GAPDH in the same RNA samples. Relative quantification was performed using the 2−ΔΔCt method (27) and expressed as fold change in arbitrary values. The primers used in the experiments are as follows: il-15 fw 5′-CATCCATCTCGTGCTACTTGTGTT-3′ and rev 5′-CATCTATCCAGTTGGCCTCTGTTT-3′; gapdh fw 5′-TCGTCCCGTAGACAAAATGG-3′ and rev 5′-TTGAGGTCAATGAAGGGGTC-3′.

### Statistical Analysis

Data are shown as the mean ± SEM. Statistical significance was assessed by Student’s t-test or one-way ANOVA for parametrical data, as indicated; Holm–Sidak test was used as a *post hoc* test; Mann–Whitney rank test and Kruskal–Wallis for non-parametrical data; and Dunn’s or Tukey’s *post hoc* tests. For multiple comparisons, multiplicity-adjusted p-values are indicated in the corresponding figures (*p 0.05, **p 0.01). Statistical analyses comprising calculation of degrees of freedom were done using SigmaPlot 11.0 and Prism 7 software.

## Results

### rAAV2-IL-15-Infected Microglia Increase the Production of IL-15, Enhancing the NK Cell Proliferation *In Vitro*

**Figure 1:**
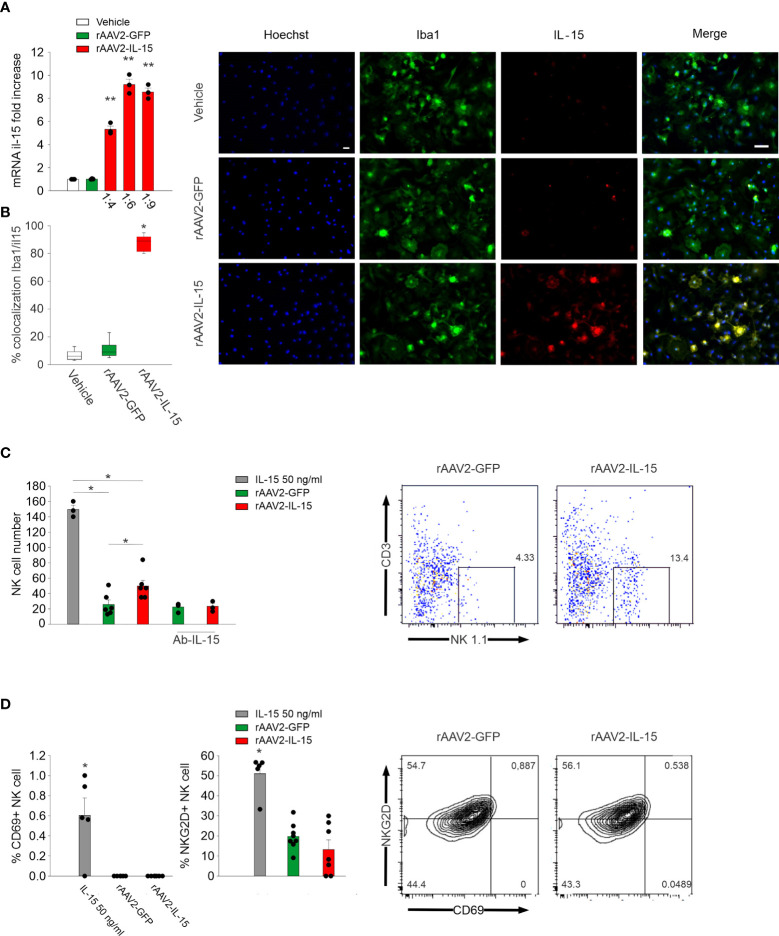
rAAV2-IL-15-infected microglia increase NK cell viability. **(A)** RT-PCR of the il-15 gene in primary microglia obtained from pups (P0–2) incubated for 7 days with vehicle, rAAV2-GFP, and rAAV2-IL-15 at different dilutions (n = 3, data are the mean ± s.e.m, **p < 0.01 one-way ANOVA). **(B)** Colocalization of IL-15- and Iba1-covered areas in primary culture of microglia treated with vehicle, rAAV2-GFP, and rAAV2-IL-15 (1:9) for 7 days (n = 6 data are the mean ± s.e.m. **p < 0.01 one-way ANOVA). For boxplots, the center line, boxes, and whiskers represent the median, inner quartiles, and the rest of the data distribution, respectively. Right: representative immunofluorescence of primary cultured microglia (stained with Iba1 in green) and IL-15 (red). Scale bar: 20 µm. **(C, D)** After 7 days of incubation with rAAV2-GFP and rAAV2-IL-15 (1:9), primary cultures of microglia were incubated with NK cells, isolated from the spleen of C57BL/6 mice, in a 1:1 ratio, in the presence of IL-15 (50 ng/ml), a specific Ab against IL-15 or vehicle; NK cell number **(C)** and CD69+ and NKG2D+ NK cells **(D)** were assessed by FACS analyses (n = 6, *p < 0.05 one-way ANOVA). Error bars show mean ± s.e.m.

At first, we analyzed the expression of IL-15 in rAAV2-infected primary microglial cultures, and their communications with NK cells *in vitro*. We incubated primary microglia with rAAV2-IL-15 for 7 days and observed an increased expression of IL-15 (both protein and mRNA, **Figures 1A, B**). Co-incubation of rAAV2-IL-15 microglia with NK cells almost doubled the number of NK cells after 72 h (NK cells with rAAV2-GFP-microglia: 25.7 ± 5 per well; NK cells with rAAV2-IL-15-microglia 50.0 ± 7 per well, n = 6 *p < 0.05 one-way ANOVA). This effect was due to the release of IL-15, because it was completely abolished in the presence of an IL-15-neutralizing antibody (**Figure 1C**). Interestingly, rAAV2-IL-15 microglia did not increase the number of NKG2D+ and CD69+ NK cells in culture (**Figure 1D**), indicating a specific effect on proliferation.

### Intranasally Delivered Microglia Enter the Brain and Reach Glioma

**Figure 2:**
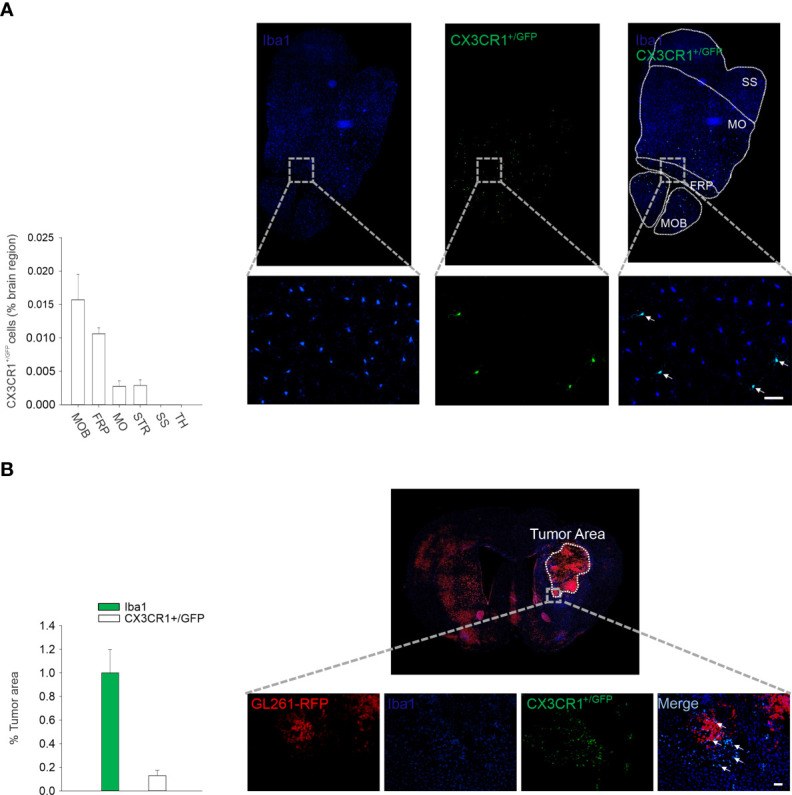
Intranasal delivered microglia reachs glioma mass. **(A)** Migration of CX3CR1^+/GFP^ microglia in the brain of C57BL/6 mice after 3 days from i.n. delivery. Right: representative immunofluorescence of Iba1 (blu) and CX3CR1^+/GFP^ cells (green) in the transversal section of mouse brain (bottom: ×20 magnification, scale bar 50 µm). MOB: main olfactory bulb; FRP: frontal pole; MO: somatomotor areas; STR: striatum; SS: somatosensory areas; TH: thalamus. Left: the mean (± s.e.m.) area of CX3CR1^+/GFP^ cells in different brain regions (as % of the area) in C57BL/6 male mice (n = 3). **(B)** Intranasal delivery of microglia (CX3CR1^+/GFP^) in GBM-bearing mice. Right: representative immunofluorescence of Iba1 (microglia/macrophage marker, in blue) and CX3CR1^+/GFP^ cells (intranasally delivered microglia expressing CX3CR1 receptor in green) in coronal sections, 17 days after GL261 implantation in mice (scale bar: 50 µm). Red: tagRFP-GL261 cells. Left: the mean (±–s.e.m.) area covered by Iba1 and CX3CR1^+/GFP^ cells in the tumor area in tagRFP-GL261-bearing mice (n = 4).

We then used microglia isolated from CX3CR1^GFP/-^ mice to express IL-15 (rAAV2-IL-15-CX3CR1^GFP/-^ microglia); these cells were then delivered through intranasal administration to glioma-bearing mice to locally increase IL-15 expression. First, we followed the route of intranasally delivered CX3CR1^GFP/-^ microglia in the brain of *wild-type* mice: data shown in **Figure 2A** report that after 3 days, GFP fluorescence was detectable in different brain structures, with a descending gradient from the point of administration (olfactory bulb). Interestingly, GFP/IBA1 double-positive cells accumulated in the tumor mass of glioma-bearing mice, representing approximately 20% of total M/M^Φ^ infiltrating the tumor (**Figure 2B**).

#### Intranasally Delivered rAAV2-IL-15 Microglia Reduce Glioma Growth and Modulate the Tumor Microenvironment *In Vivo*

**Figure 3:**
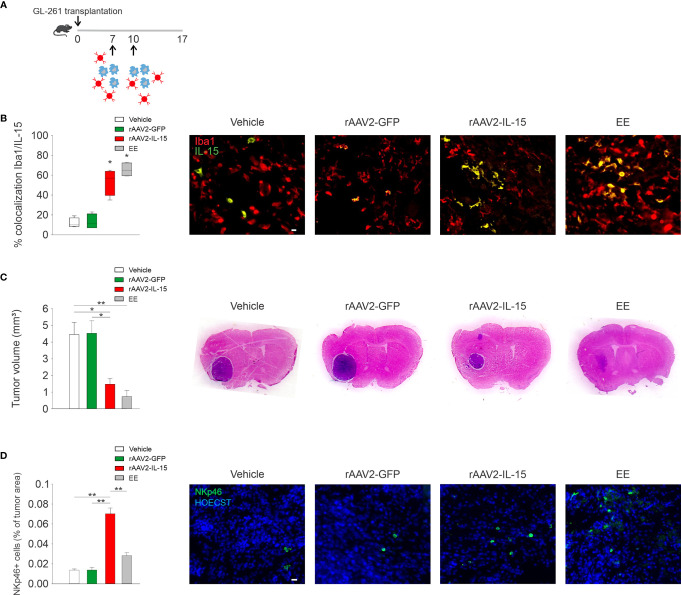
rAAV2-IL-15-microglia increase NK cell accumulation and reduce glioma volume. **(A)** Scheme of intranasal delivery of rAAV2-IL-15 microglia in GL261-transplanted mice. **(B)** Colocalization of IL-15- and Iba1-covered areas in tumor mass in mice i.n. treated with rAAV2-GFP or rAAV2-IL-15 microglia (n = 4 data are the mean ± s.e.m. *p < 0.05 Student’s t-test). For boxplots, the center line, boxes, and whiskers represent the median, inner quartiles, and the rest of the data distribution, respectively. Right: representative immunofluorescence of Iba1+ cells (red) and IL-15+ cells (green). Scale bar: 20 µm. **(C)** Tumor volume in murine GL261-bearing mice treated i.n. with vehicle, rAAV2-GFP, or rAAV2-IL-15 microglia, or exposed for 5 weeks in EE as indicated. Tumor volumes were measured 17 days after implantation of glioma cells into the striatum of C57BL/6 mice (n = 4-7, data are the mean ± s.e.m. *p < 0.05, **p < 0.01 one-way ANOVA). Right: representative coronal section of glioma-bearing mice stained with H&E. **(D)** Analyses of NKp46+ cell infiltration in tumor, expressed as % NKp46 cells in the tumor area (± s.e.m.; *p < 0.05 one-way ANOVA; n = 4 mice per condition). Representative immunofluorescence of NKp46 cells (green) 17 days after GL261 cell transplantation in mice treated i.n. with rAAV2-GFP or rAAV2-IL-15 microglia are shown on the right. Scale bar: 20 µm.

To investigate the efficacy of IL-15 produced by engineered microglia against glioma, we intranasally delivered these cells every 3 days starting 7 days after GL261 glioma cell transplantation in mice (see scheme in **Figure 3A**). We reported that mice that received rAAV2-IL-15 microglia had a higher number of IL-15/IBA1 double-positive cells in the tumor area, 17 days after glioma transplantation (**Figure 3B**). Furthermore, these mice had reduced glioma volume, similar to that observed in EE-housed mice, and in contrast with mice receiving vehicle or rAAV2-GFP microglia (n = 5–7 mice, veh 4.45 ± 0.72 mm^3^; rAAV2-GFP 4.52 ± 0.75 mm^3^; rAAV2-IL-15 1.47 ± 0.34 mm^3^; EE 0.72 ± 0.37 mm^3^) (**Figure 3C**). We previously demonstrated that EE exposure increases the accumulation of NK cells in the tumor mass with an IL-15-dependent mechanism (20). Here, we demonstrated that the delivery of rAAV2-IL-15 microglia enhances the NK cell accumulation in the tumor area, boosted also with respect to the effects of EE (**Figure 3D**).

**Figure 4:**
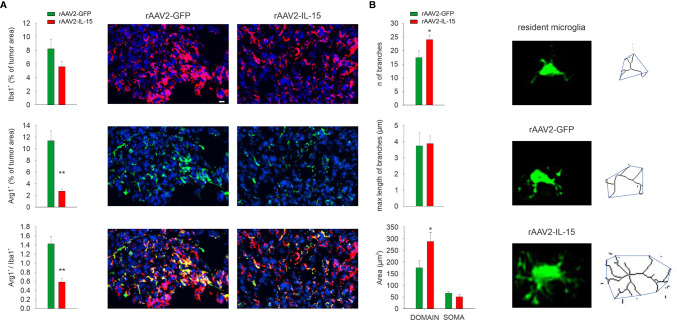
Intranasal delivery of rAAV2-IL-15-infected microglion-shaped M/M^Φ^ phenotype. **(A)** Quantification of Iba1+, Arg1+, and Iba1+/Arg1+ cells in the tumor area upon i.n. delivery of rAAV2-GFP or rAAV2-IL-15 microglia. Bars represent the mean (± s.e.m.) area expressed as percentage of total tumor areas. Representative immunofluorescence images are shown on the right (N = 4 mice per condition; **p < 0.01, *p < 0.05 Student’s *t* test). Scale bar: 20 µm. **(B)** Quantification of area of the branching, soma, and scanning domain of Iba+ cells measured by ImageJ in slices from GL261-bearing mice treated, as indicated (six slices, four mice per condition, **p = 0.0034, Student’s t-test). Below: representative images of maximum intensity projections of a confocal z-stack imaging on peritumoral area of Iba+ cells, converted to binary images and then skeletonized by the Analyze Skeleton plugin in ImageJ (green lines).

Since NK cells in glioma modulate the microglial phenotype (11, 20), we investigated the M/M^Φ^ phenotype in mice that received rAAV2-IL-15 microglia, where the NK cell number in the tumor area was increased. After 17 days from glioma transplantation, the treatment with rAAV2-IL-15-infected microglia reduced the number of Arg1+ M/M^Φ^ cells infiltrated in the tumor (a marker of pro-tumoral/anti-inflammatory M/M^Φ^ phenotype (28), (**Figure 4A**). Furthermore, M/M^Φ^ cell morphology (stained with the marker Iba1) was analyzed by two-photon microscopy, revealing alterations of cell branching and territory (i.e., mean area covered by single cells). In detail, our data show that in the tumoral region of rAAV2-IL-15 microglion-treated mice, GFP+ cells have an increased number of branches and cover a wider parenchymal region (**Figure 4B**).

## Discussion

In the last years, one big effort of biomedical research has been to try to identify the mediators of the effects of the correct lifestyle on human health. The possibility to mimic the effects of environmental signals would pave the road for new therapeutic approaches for patients suffering from non-communicable diseases (29, 30).

In addition to the known effects of the EE on brain functions and cognition (31, 32), recent evidence also demonstrated the possibility to reduce the progression of several types of cancer in murine and humanized mouse models, modulating the immune response and the pattern of cytokines released from the parenchyma and tumor cells (tumor microenvironment) (33–35). In humans, the notion that environmental stimuli can affect cancer progression has long been suspected (36). Clinical and epidemiological studies have recognized that specific psychosocial factors such as stress, chronic depression, eustress, and social support can affect the development and progression of cancer (37–39). Unfortunately, to date, despite the relevance of the issue, the translation of the environmental stimuli to pharmacological therapy for cancer patients is far to be reached.

In mouse models of melanoma and colon cancer, it was reported that living in an enriched housing environment reduces tumor growth and increases remission (19). Mechanistically, EE increased the neurothrophic brain-derived neurotrophic factor (BDNF) in the hypothalamus of tumor-bearing mice that, in turn, selectively reduced letin production *via* sympathoneural β-adrenergic signaling with direct effect on cancer cells (19). In glioma, we have recently demonstrated that 5 weeks of EE exposure reduce the tumor volume, further hampering the glioma insurgence in mouse models (20). One key molecule produced by microglial cells upon EE exposure is IL-15, which modifies the tumor microenvironment boosting the immune system to counteract glioma growth (11). IL-15 is a crucial cytokine for the development, maturation, and activation of NK cells and CD8+ T cells, with no effect on the expansion of the T regulatory cell population involved in suppressing immune responses, and thus has a potential therapeutic use in cancer immunotherapy (20, 40, 41). It has been shown that IL-15 also enhances the antitumor efficacy of the extracellular vesicles derived from NK cells, showing higher cytolytic activity against GBM (42). However, the therapeutic efficacy of IL-15 in glioma has not been tested until recently, partially due to its short half-life and low biological activity *in vivo* (43), and the systemic, non-local route of administration (44). Accordingly, a continuous and local action of IL-15 in the tumor microenvironment would be fundamental to counteracting GBM growth and progression.

In the last 10 years, few pharmacological approaches have been tested to take advantage of IL-15 for the treatment of GBM patients: a novel double-controlled oncolytic adenovirus driven by the Ki67 promoter and armed with IL-15 selectively infects and kills GBM cells, while sparing normal cells, and significantly enhances the activation of microglial cells (45). Furthermore, the administration of an IL-15 superagonist complex (ALT-803) leads to long-term survival and antitumor immune response in murine models of GBM (41).

In this paper, we investigated the possible therapeutic efficacy of the adoptive transfer of microglia, modified with the rAAV vector carrying IL-15, to mimic the EE effects on glioma. The possibility to exploit microglial cells intranasally delivered as a source of IL-15 directly in the tumor mass could be an attractive alternative for GBM patient treatment, due to the high potential for clinical translatability, as one can obtain the microglia starting from patients’ iPSCs, as recently reported (46). The local delivery of cells as therapeutic carriers against glioma has produced encouraging results but encounters obstacles with regard to the repeatability and invasiveness of administration. Intranasal delivery of cells could overcome these obstacles as a non-invasive and easily repeatable route of administration (47).

Here, we demonstrated that the infection of microglia with an AAV-serotype 2 carrying IL-15 functionally induces the release of IL-15. AAV are considered optimal for human gene therapy as they are small and non-replicative, can transduce dividing and nondividing cells, are non-pathogenic in humans, and can determine long-lasting changes in gene expression. Importantly, AAV have been used to target several cell types, including microglia (24, 48).

In our studies, the rAAV2-IL-15 microglia activated the interplay with NK cells both *in vitro* and *in vivo*. In particular, these infected microglia increased the number of NK cells in coculture, without affecting their activation state. This could be due to the dose-dependent effect of IL-15: in the *in vitro* system, the rAAV2-IL-15 microglia produce a concentration of IL-15 with effects on proliferation and viability, without reaching the higher doses needed to activate the NK cells (49, 50). We recently reported that microglial production of IL-15, in glioma bearing in EE-housed mice, recruits IFN-γ+ NK cells, with effects on glioma growth (20). Attempting to reproduce the effect of EE on glioma, we showed that intranasally delivered rAAV2-IL-15 microglia reach glioma and increase NK cell accumulation in glioma-bearing mice, highlighting the fundamental role of IL-15 in the tumor core to boost the immune reaction.

Since recruited NK cells could revert the GBM microenvironment instructing microglia toward a pro-inflammatory/antitumoral phenotype (11), we investigated the M/M^Φ^ state in tumor mass. We showed that rAAV2-IL-15 microglia consistently modulate the M/M^Φ^ state with the reduction of arginase levels and an increased number of branches and covered the parenchymal region, suggesting the switch in an antitumoral phenotype (11, 28). Further, these data suggest that the recruited NK cells in the tumor core are activated, thus explaining the modulation of the M/M^Φ^ phenotype (11).

Altogether, our results demonstrated that i.n. delivery of IL-15-engineered microglia is able to reproduce the effect of EE on NK cell and tumor volume in glioma mouse models. These preclinical data support the use of rAAV2-IL-15-infected microglia as a tool to vehicle and translate the EE signals inside the tumor mass and offer a new perspective to use them as Trojan horses to modify the tumor microenvironment.

## Data Availability Statement

The original contributions presented in the study are included in the article/supplementary material. Further inquiries can be directed to the corresponding authors.

## Ethics Statement

The animal study was reviewed and approved by the Italian Ministry of Health in accordance with the guidelines on the ethical use of animals from the European Community Council Directive of September 22, 2010 (2010/63/EU).

## Author Contributions

AM: performed most of the experimental work and wrote the paper. GC: contributed to many experimental activities for mouse manipulation. GB: performed FACS analyses. NC: sequenced and provided adeno-associated viral particles and wrote the paper. CP: sequenced and provided adeno-associated viral particles and wrote the paper. AS: supervised the experiments on NK cells. CL: supervised all the experimental work and wrote the manuscript. SG: ideated and supervised all the experimental work and wrote the paper. All authors contributed to the article and approved the submitted version.

## Funding

This work was supported by AIRC 22329 2018 to SG and PRIN 2017, AIRC 2019, Ministero della Salute RF2018 to CL.

## Conflict of Interest

The authors declare that the research was conducted in the absence of any commercial or financial relationships that could be construed as a potential conflict of interest.

## Publisher’s Note

All claims expressed in this article are solely those of the authors and do not necessarily represent those of their affiliated organizations, or those of the publisher, the editors and the reviewers. Any product that may be evaluated in this article, or claim that may be made by its manufacturer, is not guaranteed or endorsed by the publisher.
